# Disease‐Derived Tau Seeds Induce Aggregation and Pathway Disruptions in a Cellular Model

**DOI:** 10.1002/alz70855_106344

**Published:** 2025-12-24

**Authors:** Tomas Kavanagh, Kaleah Balcomb, Aysha Strobbe, He‐Shyan Balnave, Diba Ahmadi Rastegar, Evgeny Kanshin, Beatrix Ueberheide, Glenda M Halliday, Eleanor Drummond

**Affiliations:** ^1^ The University of Sydney, Sydney, NSW, Australia; ^2^ NYU Grossman School of Medicine, New York, NY, USA

## Abstract

**Background:**

Tau aggregation is a hallmark of tauopathies, including Alzheimer's disease (AD), progressive supranuclear palsy (PSP), Pick's disease (PiD). While tau aggregation is common amongst these diseases, biochemical differences—such as aggregate composition (3R, 4R, or 3R+4R tau) and post‐translational modifications—may influence toxicity. Current models typically rely on tau overexpression or use synthetic fibrils which lack the biochemical complexity of disease‐derived tau. To address this limitation, we developed a tau seeding model without overexpression, enabling broader studies of tau interactions, toxicity, and therapeutic interventions in any cell‐type.

**Method:**

Tau aggregates were purified from AD, PiD, PSP, and cognitively normal controls (*n* = 3) brains using biochemical fractionation and precipitation and characterized by mass spectrometry. Normalized quantities of tau seeds were seeded onto SH‐SY5Y, M03.13, U‐87 and U‐118 cells to induce tau aggregation. Aggregates were quantified using wide‐field and confocal imaging. Cytotoxicity assays and pathway markers were used to assess tau toxicity and its impact on autophagy, lysosomes and mitochondria.

**Result:**

Disease‐derived tau seeds retained disease specific properties and were successfully used to induce tau aggregation across multiple cell lines in a titratable manner. Mass‐spectrometry confirmed expected isoform biases, including elevated 4R tau in AD and PSP and identified APP enrichment in AD preparations. Mass spectrometry detected some contaminating proteins, but they were also present in similar abundance in control tau preparations. While seeding efficiency varied by cell type, PiD‐derived tau seeds showed the highest seeding capacity, averaging 1.4 tau aggregates per cell compared to 0.6 for AD in SH‐SY5Y cells. Optimized tau concentrations resulted in minimal short‐term cytotoxicity. However, tau seeds induced distinct pathway‐specific disruptions, with PiD‐derived seeds doubling p62 puncta formation compared to control‐derived tau.

**Conclusion:**

This approach provides a robust model for studying tau pathology in a disease‐specific context. By eliminating tau overexpression, it provides a more physiologically relevant platform to investigate tau interactions and assess potential therapeutic strategies targeting aggregation and toxicity.

